# Wide Distribution of Genes for Tetrahydromethanopterin/Methanofuran-Linked C1 Transfer Reactions Argues for Their Presence in the Common Ancestor of Bacteria and Archaea

**DOI:** 10.3389/fmicb.2016.01425

**Published:** 2016-09-13

**Authors:** Ludmila Chistoserdova

**Affiliations:** Department of Chemical Engineering, University of WashingtonSeattle, WA, USA

**Keywords:** methanogenesis, methylotrophy, tetrahydromethanopterin, methanofuran, C1 transfer, evolution

In this opinion article, I wish to highlight the fact that reactions linked to tetrahydromethanopterin (H_4_MPT) and methanofuran (MF), the ones involved in methanogenesis as well as in methylotrophy, are much more widespread among both Bacteria and Archaea than originally thought. While, over the past two decades, databases of the respective genes have been steadily growing and expanding to include novel, divergent sequences, belonging to a variety of taxa, somehow a view still prevails of the limited distribution of these genes, along with an evolutionary scenario in which genes for the methanogenesis pathway were horizontally transferred from Euryarchaea into Proteobacteria (Graham et al., 2000; Gogarten et al., 2002; Boucher et al., 2003; Braakman and Smith, 2012; Arnold, 2015). The two main arguments originally used to support this scenario were (1) the limited distribution of the H_4_MPT/MF-dependent pathway in the bacterial domain of life, and (2) the low probability of the respective genes being lost in most bacterial lineages (Boucher et al., 2003). However, these arguments can be easily refuted in the light of the current knowledge. In Figure 1, I utilize the recently constructed universal tree of life (Hug et al., 2016), to map the taxa in which at least some of the genes for the H_4_MPT/MF-dependent C1 transfers are recognized. Among the Archaea, these include, in addition to the well-characterized methanonogens or methane oxidizers, members of Euryarchaeota not known for a methanogenic life style (Thermoplasmatales, Hadesarchaea; Baker et al., 2016), members of Crenarchaeota (Thermoproteales, *Ignisphaera, Ingnispaeroid*; Göker et al., 2010; Jay et al., 2016), Bathyarchaeota (Evans et al., 2015; Lazar et al., 2016), and Thorarchaeota (Seitz et al., 2016). Among the Bacteria, genes for the H_4_MPT/MF-dependent reactions have been identified, beside Alpha-, Beta-, and Gammaproteobacteria (Vorholt et al., 1999), in the genomes of Planctomycetes (Chistoserdova et al., 2004; Chistoserdova, 2013), Deltaproteobacteria, Firmicutes, Actinomycetes, Synergistetes, Chloroflexi (Brown et al., 2011 and unpublished genomes available through the NCBI), as well as in the Candidate phylum NC10 (Ettwig et al., 2010). This wide distribution across the tree of life (Figure 1), along with great sequence divergence for the genes in question (Chistoserdova, 2013; Evans et al., 2015; Spang et al., 2015) support a scenario of a long evolution within both Archaea and Bacteria, and point to the emergence of these reactions in early life, before Bacteria and Archaea have branched apart.

**Figure 1:**
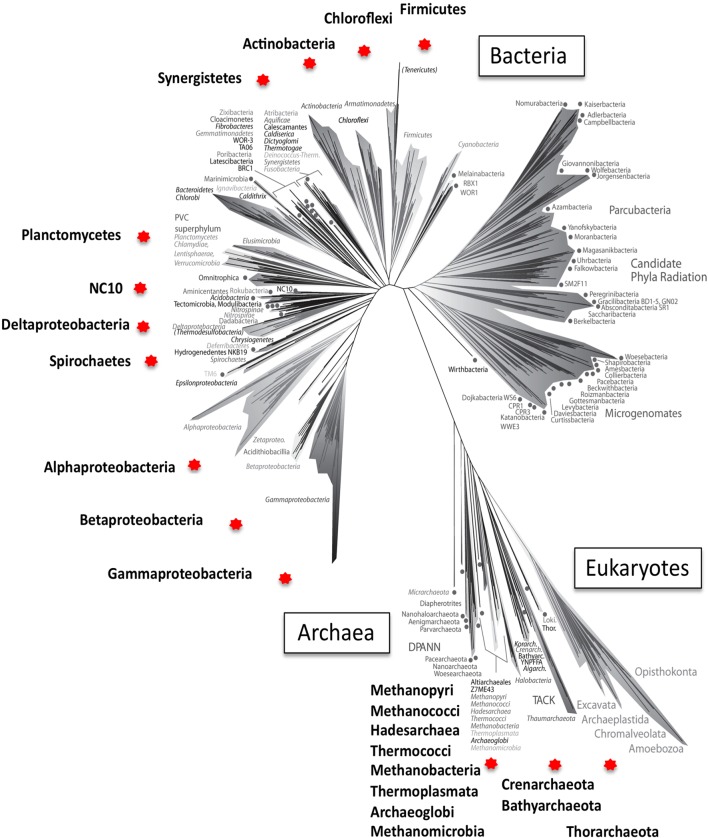
**A universal tree of life reconstructed by Hug et al. (2016), adapted (with authors' permission) to highlight the points of this Opinion Article**. Hug et al. used 16 concatenated ribosomal protein sequences from 3083 organisms representing major known lineages at the Phylum level, with the exception of Proteobacteria, which are represented at the Class level, as these are not monophyletic. 1011 of the genomes utilized, reconstructed from metagenomes, represent uncultivated phyla. Phyla (Classes) containing representatives encoding enzymes for the entire or a partial set of the H_4_MPT/MF-linked reactions (Chistoserdova, 2011) are denoted by red stars, and names are magnified. Note, that the top right area of the tree is represented entirely by organisms without cultivated representatives, possessing small genomes, with evidence of restricted metabolic capacities and suggesting symbiotic lifestyle (references in Hug et al., 2016). Genes for the H_4_MPT/MF-linked functions were likely lost from these lineages, along with other functions such as the complete tricarboxylic acid cycle, amino acid biosynthesis, etc. (Hug et al., 2016).

As to the second argument, of a low probability of the massive loss of genes in question, we now have multiple examples to support occurrence of such events, in major microbial taxa. One example is the methylotrophs of the *Methylophilaceae* family that are represented by species from soils or sediments, possessing larger genomes, all encoding the H_4_MPT/MF-dependent functions (Beck et al., 2014), and by planktonic species, which, while closely related to the former, posses much smaller genomes, not encoding any H_4_MPT/MF-linked functions, due to the proposed genome reduction (Giovannoni et al., 2008; Huggett et al., 2012; Jimenez-Infante et al., 2015; Salcher et al., 2015). One dramatic example is presented by the genomes of the *Nitrosococcus* species. While the genomes of *Nitrosococcus halophilus* and *Nitrosococcus wardiae* encode the entire complement of the H_4_MPT/MF-dependent C1 transfer reactions essential for formaldehyde oxidation, along with a methanol dehydrogenase (Campbell et al., 2011; Wang et al., 2016; M. G. Klotz, personal communication), suggesting a potential in methanol oxidation, the closely related species *Nitrosococcus watsonii* and *Nitrosococcus oceani* (Klotz et al., 2006; Campbell et al., 2011; Wang et al., 2016) possess conserved gene clusters that lack some of the key genes in the pathway, and they also lack methanol dehydrogenase genes, suggesting relatively recent loss of function. Another example of variable pathway presence is the *Burkholderia* species, whose genomes have been extensively sampled. Of the 619 genomes available through the IMG database (https://img.jgi.doe.gov/) 74 possess the genes in question (12% of total genomes). However, the *Burkholderia* database is heavily skewed toward the pathogenic species *Burkholderia mallei* and *Burkholderia pseudomallei* of a specialized life style typically resulting in genome reduction through gene loss (Ochman and Moran, 2001; Moran, 2002; Song et al., 2014). If these pathogenic species are excluded from the analysis, then 28% of *Burkholderia* species are positive for encoding the complete pathway for formaldehyde oxidation, with gene sequences and gene clustering patterns highly conserved, indicative of vertical inheritance. Interestingly, 13 of the genomes encode a second, phylogenetically distinct set of genes for H_4_MPT/MF-dependent C1 transfer reactions, suggesting that these may have been laterally transferred to a sub-lineage of *Burkholderia* (Chistoserdova, 2011 and recent observations). Similar observations on the presence/absence of H_4_MPT/MF-C1 transfer genes can be made for Archaea of different phylogenetic positions. Some of the Thermoplasmatales genomes encode the H_4_MPT/MF-linked functions and some do not. Likewise, some *Ignisphaera* genomes encode these functions and some do not, suggesting recent gene losses. More ancient, lineage-specific gene losses are also apparent. One example is the *fwdD* gene homolog (encoding a putative subunit of the formyltransferase/hydrolase complex) that is maintained in Archaea, Synergistetes, Firmicutes, and Candidate phylum NC10, but is not present in either Planctomycetes or Proteobacteria (Chistoserdova, 2013). Another example is the Afp protein that is encoded in the genomes of Archaea, Synergistetes, Firmicutes, and most Proteobacteria (Chistoserdova, 2013). However, the respective gene is not recognized in the Planctomycete genomes or in the genomes of some Proteobacteria. Its non-homologous substitution, DmrA, has been identified in the *Methylobacterium* species (Alphaproteobacteria; Marx et al., 2003; Caccamo et al., 2004; Vuilleumier et al., 2009). A third example is a methanofuran biosynthesis gene *mfnD* (Wang et al., 2014; also known as *orf1*, Kalyuzhnaya et al., 2005). This gene is also not recognizable in *Methylobacterium* genomes. As methanofuran can be measured in these bacteria (Hemmann et al., 2016), a non-homologous substitution must be present.

Overall, with the growing genomic databases and with the increasing representation of environmental versus pathogenic microbes, the distribution of the H_4_MPT/MF-linked functions appears to be much less sparse than previously assumed, and these functions are especially frequently present in species that are subject to selective pressure for their maintenance (methanogens and methylotrophs, for example). The recent models of the evolution of metabolic pathways in living organisms also support spotty distribution of genes for ancient pathways (Nitschke and Russell, 2013), and gene loss in general is considered as a prominent evolutionary force in shaping genomic contents of extant organisms (Koonin and Yutin, 2014; Albalat and Cañestro, 2016).

The expanded diversity within the domain of Archaea, with many lineages encoding the H_4_MPT/M-linked reactions, even if their specific roles may remain elusive in the novel and uncultivated species (Evans et al., 2015; Mwirichia et al., 2016; Seitz et al., 2016) also questions the original proposal of the ancestral position of the methanogens in terms of the emergence of the H_4_MPT/MF-linked reactions (Graham et al., 2000; Gogarten et al., 2002; Boucher et al., 2003). It seems more likely now that the emergence of these reactions predated superphylum radiation within Archaea.

In conclusion, the current evidence supports neither emergence of the H_4_MPT/MF-linked functions in the Euryarchaeota, nor their transfer from Euryarchaeota into Bacteria. Instead, the recent data suggest an early evolution of the respective genes/pathways in the ancestor of both Bacteria and Archaea. Intriguingly, some of the genes in question are present in members of “Thorarchaeota,” a recently identified Candidate phylum, closely related to members of another newly proposed phylum “Lokiarchaeota.” Both are proposed to be monophyletic with Eukaryota (Koonin, 2015; Spang et al., 2015; Seitz et al., 2016), suggesting that the H_4_MPT/MF-linked functions may have also been present in early Eukaryotes, thus placing these functions with the last universal common ancestor (LUCA) of life on Earth. Indeed, the recent reconstruction of the physiology and habitat of LUCA suggests that it possessed at least some of the genes for H_4_MPT/MF-C1 transfers and points to the importance of the C1 metabolites in early life (Weiss et al., 2016). The pathway likely predated both methylotrophy and methanogenesis, but it has likely served as an enabling block in the evolution of both. While the biochemistry of methanogenesis is absolutely reliant on the H_4_MPT/MF-dependent reactions, the biochemistry of methylotrophy does not, and thus it is not entirely clear why so many methylotrophs maintain the pathway (Chistoserdova, 2011). The role of the H_4_MPT/MF-dependent reactions in non-methanogenic or non-methylotrophic species still remains enigmatic.

## Author contributions

The author carried out literature and BLAST searches, made phylogenetic inferences, conceived, and wrote the manuscript.

### Conflict of interest statement

The author declares that the research was conducted in the absence of any commercial or financial relationships that could be construed as a potential conflict of interest.
